# Immune Checkpoint Inhibitor-Associated Cardiovascular Toxicity in Melanoma: Current Evidence and Practical Clinical Management

**DOI:** 10.3390/cancers18183041

**Published:** 2026-09-19

**Authors:** Andrea Bottardi, Gabriele Busin, Lam Nguyen, Paolo Del Fiore, Giorgia Alberti, Andrea Danese, Michele Nori, Raimondo Pittorru, Fortunato Cassalia

**Affiliations:** 1 Department of Cardiology, Clinique Saint George, 2 Avenue de Rimiez, 06105 Nice, France; 2 School of Medicine and Surgery, Center for Medical Sciences (CISMed), University of Trento, 38122 Trento, Italy; 3 Department of Medicine and Surgery, Sapienza University of Rome, 00185 Rome, Italy; 4 Soft-Tissue, Peritoneum and Melanoma Surgical Oncology Unit, Veneto Institute of Oncology IOV-IRCCS, 35128 Padova, Italy; fortunato.cassalia@iov.veneto.it; 5 Department of Cardiology, University of Verona, 37134 Verona, Italy; giorgia.alberti@univr.it; 6 Unit of Dermatology, Department of Integrated Medical and General Activity, University of Verona, 37134 Verona, Italy; andrea.danese_02@studenti.univr.it; 7 Unità Operativa Complessa (UOC) Cardiologia, Ospedale di Arzignano–Montecchio, AULSS 8 Berica, 36071 Arzignano, Italy; michele.nori@aulss8.veneto.it; 8 Division of Cardiology, Department of Cardiac, Thoracic, Vascular Sciences and Public Health, University of Padua, 35128 Padua, Italy; raimondo.pittorru@studenti.unipd.it; 9 PhD Program in Translational Specialized Medicine “G.B. Morgagni”, Curriculum in Cardiovascular Sciences, University of Padua, 35128 Padua, Italy

**Keywords:** melanoma, immune checkpoint inhibitors, cardiotoxicity, myocarditis, cardio-oncology, immune-related adverse events, cardiovascular toxicity, cardiovascular surveillance, immunotherapy, melanoma treatment

## Abstract

Immune checkpoint inhibitors (ICIs) have transformed the therapeutic landscape of advanced melanoma, significantly improving long-term survival. Nevertheless, these therapies may induce immune-related cardiovascular adverse events, which are uncommon but potentially life-threatening. Myocarditis is the best-characterized and most severe manifestation, although a broad spectrum of cardiovascular complications—including pericardial disease, arrhythmias, conduction abnormalities, heart failure, Takotsubo syndrome, and vascular events—has increasingly been recognized. Emerging evidence suggests that melanoma itself and dual immune checkpoint blockade may confer a higher risk of cardiotoxicity than other malignancies or single-agent immunotherapy. This narrative review summarizes current evidence regarding the epidemiology, pathophysiological mechanisms, clinical presentation, diagnostic evaluation, cardiovascular surveillance, and management of ICI-associated cardiotoxicity in patients with melanoma. Diagnosis relies on integrating clinical findings with electrocardiography, cardiac biomarkers, echocardiography, cardiac magnetic resonance imaging, and, in selected cases, endomyocardial biopsy. Prompt interruption of ICI therapy, early administration of high-dose corticosteroids, and escalation to additional immunosuppressive therapies in refractory cases remain the cornerstone of treatment. As the use of ICIs continues to expand, improving cardiovascular risk stratification and implementing evidence-based surveillance strategies will become increasingly important. A multidisciplinary cardio-oncology approach is essential to ensure early diagnosis, optimize cardiovascular outcomes, and preserve the anticancer efficacy of immunotherapy.

## Simple Summary

Immune checkpoint inhibitors (ICIs) have revolutionized the treatment of advanced melanoma, leading to substantial and durable improvements in patient survival. However, these agents may also trigger immune-related cardiovascular toxicities, which, although uncommon, can be severe and potentially fatal. Myocarditis is the most feared complication because of its rapid onset and high mortality, particularly in patients receiving combination immunotherapy. Other manifestations include pericardial disease, arrhythmias, conduction disorders, heart failure, Takotsubo syndrome, and vascular complications. Early recognition, appropriate cardiovascular surveillance, and prompt multidisciplinary management are essential to improve clinical outcomes while preserving the benefits of anticancer treatment. This narrative review summarizes current evidence on the epidemiology, pathophysiology, diagnosis, surveillance, and management of ICI-associated cardiotoxicity in melanoma, providing a practical overview for oncologists, cardiologists, dermatologists, and other clinicians involved in the care of melanoma patients.

## 1. Introduction

**Figure 1:**
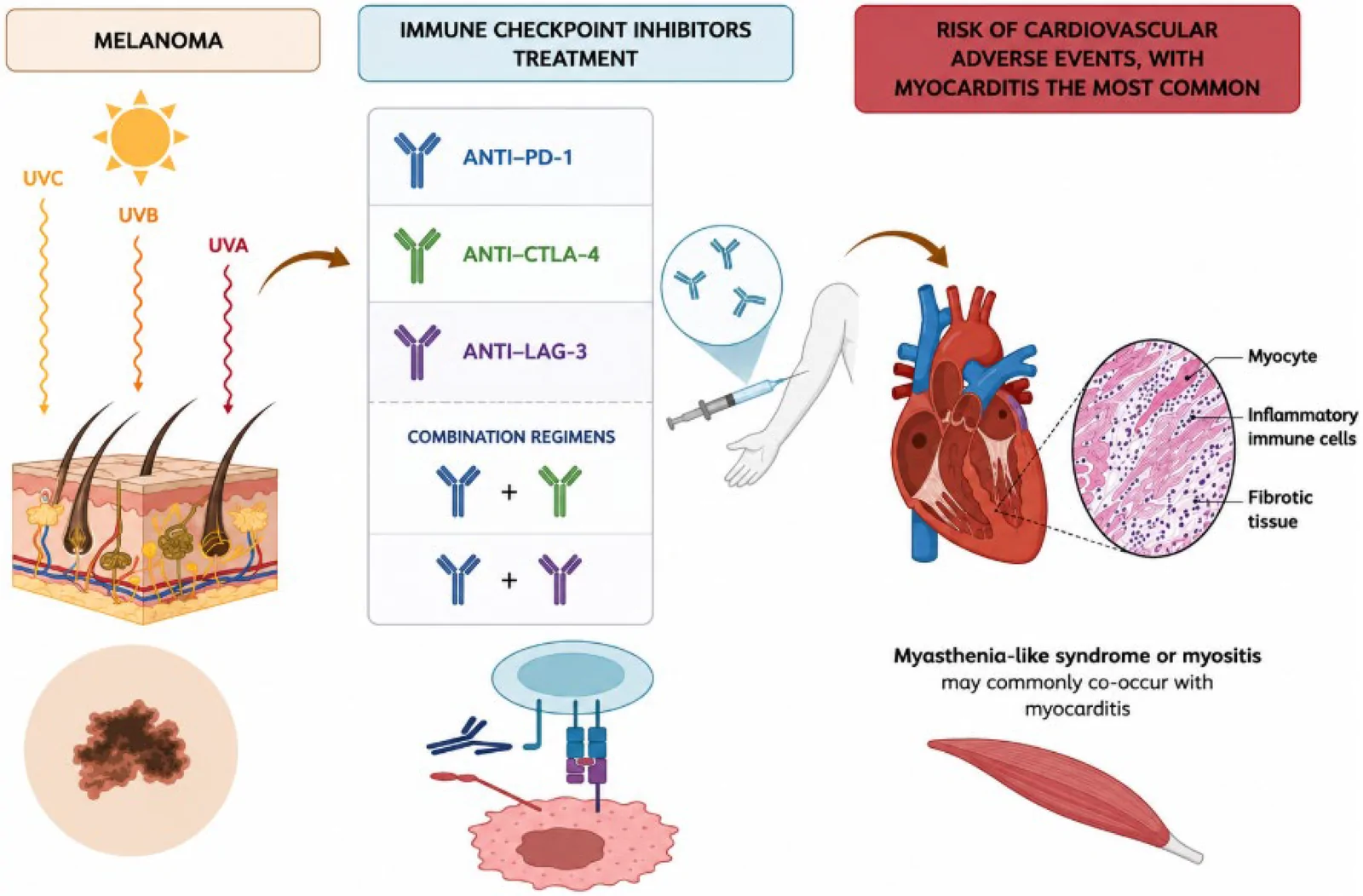
**Central Illustration: Clinical continuum from melanoma treatment to cardiovascular immune-related adverse events.** It depicts the clinical continuum from melanoma treatment with immune checkpoint inhibitors to cardiovascular immune-related adverse events. It highlights myocarditis as the most clinically relevant phenotype and emphasizes its potential association with myocardial inflammation, myositis, and myasthenia-like overlap syndromes. This figure was realized with BioRender.

Melanoma is an aggressive malignancy for which immune checkpoint inhibitors (ICIs) have produced substantial and durable improvements in survival. By blocking programmed cell death protein 1 (PD-1), programmed death-ligand 1 (PD-L1), or cytotoxic T-lymphocyte-associated protein 4 (CTLA-4), ICIs restore antitumor T-cell activity but may also disrupt immune tolerance and cause immune-related adverse events (irAEs). Although cardiovascular immune-related adverse events are less common than dermatologic, gastrointestinal, or endocrine toxicities, they may cause rapidly progressive and potentially life-threatening complications, including fulminant myocarditis, myopericarditis, cardiac dysfunction, acute heart failure, malignant arrhythmias and conduction disturbances, Takotsubo syndrome, and acute myocardial infarction, often necessitating interruption or permanent discontinuation of ICI therapy. Melanoma deserves particular attention because combination immune checkpoint blockade is frequently used, and recent epidemiological studies have identified melanoma as an independent risk factor for ICI-associated myotoxicity. This review summarizes the mechanisms, epidemiology, clinical presentation, diagnostic evaluation, surveillance, management, and outcomes of ICI-associated cardiovascular toxicity in patients with melanoma [1,2,3,4]. The clinical continuum from melanoma treatment to cardiovascular immune-related adverse events is illustrated in Figure 1.

This narrative review was based on a literature search conducted in August 2026 using PubMed/MEDLINE, Embase, and Scopus, covering publications from January 2015 to August 2026. The search was complemented by manual screening of the reference lists of relevant articles, and a limited number of seminal earlier publications were included when necessary to describe the mechanistic background. The principal search terms included combinations of “immune checkpoint inhibitor”, “melanoma”, “cardiotoxicity”, “myocarditis”, “immune-related adverse events”, “cardio-oncology”, and “cardiovascular surveillance”. Original clinical and experimental studies, pharmacovigilance and registry analyses, systematic reviews, and the most recent international guidelines and consensus documents were selected according to their relevance to the scope of this narrative review. Only articles published in English were considered.

## 2. Immune Checkpoint Inhibitors in the Current Treatment of Melanoma

ICIs are monoclonal antibodies that restore antitumor T-cell activity by blocking inhibitory immune pathways. In melanoma, the principal classes are antibodies targeting PD-1, including nivolumab and pembrolizumab; CTLA-4, represented by ipilimumab; and LAG-3, represented by relatlimab [5]. These agents may be administered alone or in combination according to disease stage, tumor characteristics, and patient-related factors.

After complete surgical resection, anti-PD-1 monotherapy with pembrolizumab or nivolumab can be administered as adjuvant treatment; that is, postoperative therapy aimed at reducing the risk of recurrence in patients with high-risk stage IIB–IIC or stage III melanoma [5]. The efficacy of this approach in improving recurrence-free and distant metastasis-free survival has been demonstrated in the KEYNOTE-716 and CheckMate 76K trials in stage IIB–IIC melanoma [6,7] and in the KEYNOTE-054 and CheckMate 238 trials in stage III disease [8,9]. In patients with clinically detectable, resectable stage III melanoma, ICIs may also be administered as neoadjuvant therapy before surgery. This strategy treats micrometastatic disease early, while tumor tissue and tumor-specific lymphocytes are still present, and allows the pathological response to guide postoperative treatment. In SWOG S1801, perioperative pembrolizumab improved event-free survival compared with surgery followed by adjuvant pembrolizumab alone [10]. The phase III NADINA trial subsequently demonstrated superior event-free survival with neoadjuvant ipilimumab plus nivolumab followed by surgery and response-directed adjuvant therapy compared with surgery followed by nivolumab [11]. For unresectable stage III or metastatic stage IV melanoma, immunotherapy is a cornerstone of systemic treatment. Available options include nivolumab or pembrolizumab monotherapy, combined nivolumab-ipilimumab, and combined nivolumab–relatlimab [5]. CheckMate 067 demonstrated durable long-term survival with nivolumab-based therapy [12], whereas RELATIVITY-047 showed improved progression-free survival with dual PD-1/LAG-3 blockade compared with nivolumab alone [13]. Combination regimens may increase antitumor activity but also expose patients to greater immune-related toxicity. This benefit–risk balance is particularly relevant to cardiovascular safety because dual-checkpoint blockade, especially combined PD-1 and CTLA-4 inhibition, is associated with a higher risk of myocarditis and other immune-mediated cardiovascular adverse events than anti-PD-1 monotherapy [1,2,3].

## 3. Epidemiology of Cardiotoxicity in Melanoma Patients on ICIs

**Figure 2:**
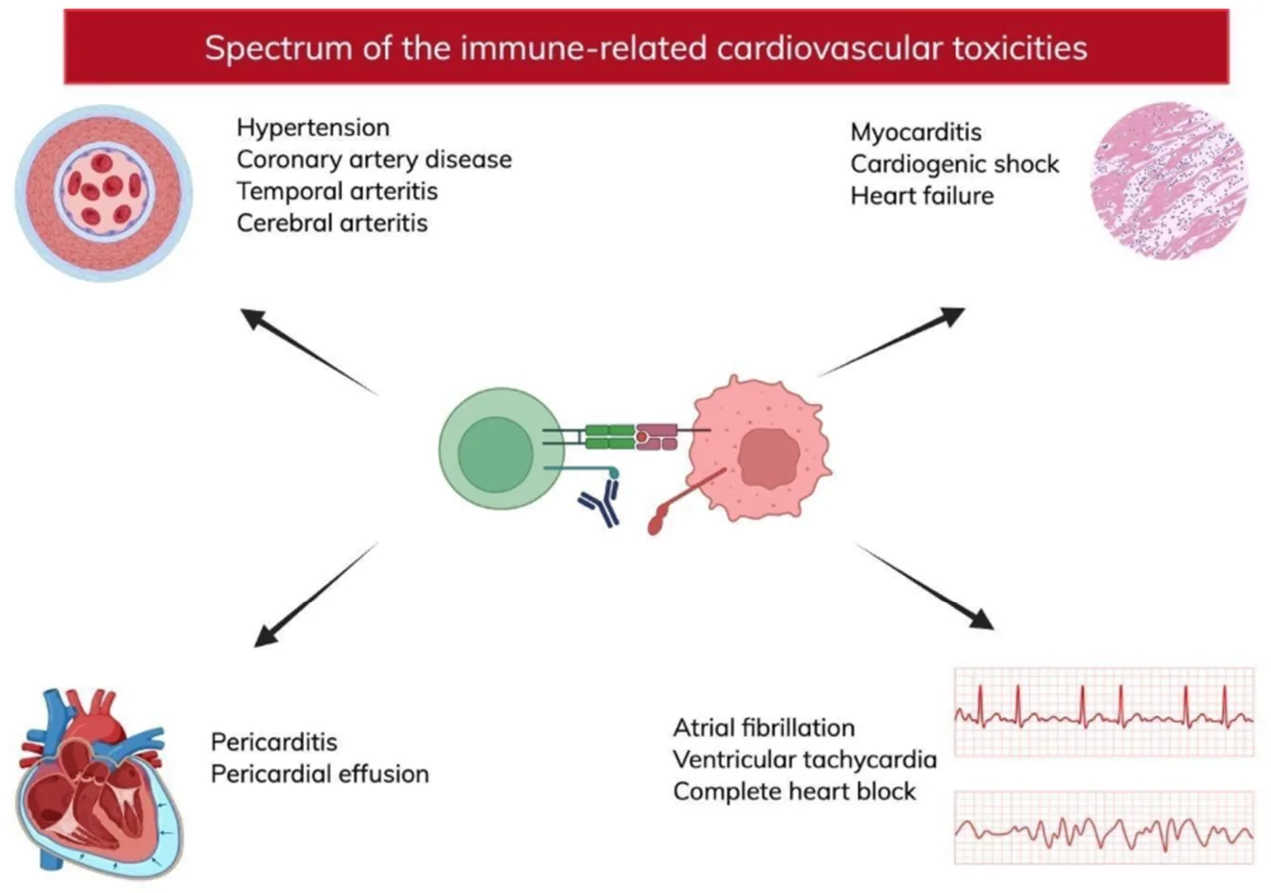
**Spectrum of immune-related cardiovascular toxicities associated with immune checkpoint inhibitors.** The figure illustrates the principal cardiovascular phenotypes of ICI therapy, including myocarditis, heart failure, cardiogenic shock, pericardial disease, arrhythmias and conduction disturbances, hypertension, coronary artery disease, and immune-mediated vascular inflammation. This figure was realized with BioRender.

Cardiotoxicity in patients receiving ICIs, particularly those with melanoma, remains a relatively rare but serious complication. The main forms of irCAEs (immune-related cardiac adverse events) include myocarditis and non-inflammatory syndromes (Figure 2). As the population eligible for ICI therapy continues to expand, the absolute burden of ICI-associated myocarditis is expected to rise. Early case series reported mortality approaching 50% [14]; however, more recent multicentre registries suggest substantially lower mortality owing to earlier diagnosis and systematic surveillance [4]. Similarly, an analysis of the World Health Organization pharmacovigilance database reported a case-fatality rate of up to 33% [15]. The incidence of ICI-induced myocarditis in clinical trials has been reported to range from 0.04% to 1.14% [16].

Non-inflammatory syndromes include Takotsubo syndrome, non-inflammatory heart failure or left ventricular dysfunction, and post-MI heart failure. In the largest multicentre registry to date (n = 748, 17 countries), 30-day cardiomyotoxic death was 13% and overall death was 17%. In a French nationwide study of over 172,000 ICI-treated patients, 1-month fatality among myotoxicity cases was 20.3%. Severe myocarditis typically occurs early after ICI initiation (median 30 days; IQR 18–60 days). Moreover, ICI-related cardiac adverse events more commonly involve men and patients older than 65 years with comorbidities [17]. The most important conditions associated with the onset of irCAEs include dual ICI therapy, combination with other cardiotoxic therapies and the presence of underlying cardiovascular risk factors [18,19,20].

Importantly, melanoma itself has been identified as an independent risk factor for ICI-related myotoxicity. In the French nationwide study, melanoma was associated with a sub-distribution hazard ratio of 2.41 (95% CI, 2.06–2.83) for developing ICI myotoxicity. Similarly, in a global real-world cohort of over 130,000 ICI-treated patients from the TriNetX network, melanoma was independently associated with myocarditis (HR 1.52; 95% CI, 1.25–1.86; *p* = 0.01) on multivariate analysis [3,4]. One biologically plausible explanation is cross-reactive antitumor immunity, whereby tumor-directed T-cell clonotypes may recognize shared or structurally related antigens expressed in melanoma and cardiac tissue. However, although shared T-cell receptor clonotypes have been identified in tumor, myocardium, and skeletal muscle in patients with ICI-associated myocarditis, a causal relationship between this mechanism and the higher epidemiological risk observed in melanoma has not yet been established.

It is noteworthy that the incidence of cardiotoxicity increases notably in patients receiving combination therapy (e.g., anti-CTLA-4 and anti-PD-1 inhibitors) as compared to those on monotherapy [21]. A systematic review and meta-analysis of clinical trial data reported an all-grade myocarditis incidence of 0.97% (95% CI, 0–2.40%) in patients treated with nivolumab 3 mg/kg plus ipilimumab 1 mg/kg, compared to 0.23% (95% CI, 0–0.42%) with PD-1/PD-L1 monotherapy (*p =* 0.001) [22]. These findings are supported by the French nationwide study, in which combination ICI therapy was independently associated with a higher risk of myotoxicity (sHR 2.44; 95% CI, 1.99–2.98). Collectively, these data suggest that dual checkpoint inhibition enhances the likelihood of immune-mediated cardiotoxicity, likely due to a more profound and widespread activation of the immune system. Given that combination nivolumab-ipilimumab is a standard first-line regimen for advanced melanoma, the convergence of a high-risk regimen with a high-risk tumor type underscores the particular importance of cardiovascular vigilance in this population. Melanoma patients who have pre-existing cardiovascular conditions, such as coronary artery disease or hypertension, are at an increased risk of developing cardiotoxicity. This is especially concerning given that many patients with advanced melanoma are older adults with a higher prevalence of cardiovascular comorbidities [1,2,3,4,23].

## 4. Mechanism of ICI-Induced Cardiotoxicity in Melanoma Patients

ICI-associated cardiotoxicity results from disruption of immune checkpoints that are essential to peripheral self-tolerance. Although myocarditis is the best-characterized phenotype, checkpoint inhibition can affect the myocardium, conduction system, pericardium, vasculature, and skeletal muscle through partially overlapping immune pathways. The evidence is strongest for T-cell-mediated myocardial injury, whereas the contribution of humoral immunity, complement, and other innate immune effectors remains less firmly established [24].

### 4.1. T-Cell-Mediated Myocardial Injury

The clearest mechanistic evidence supports loss of tolerance to cardiac antigens and expansion of autoreactive T cells. In the first detailed report of fulminant myocarditis after combined nivolumab and ipilimumab in patients with melanoma, T-cell receptor sequencing identified shared high-frequency clonotypes in tumor, myocardium, and skeletal muscle, suggesting that antitumor T cells may cross-recognize antigens expressed in striated muscle [25]. Subsequent experimental work identified alpha-myosin heavy chain (alpha-MyHC) as a leading cardiac autoantigen. Axelrod et al. demonstrated expansion of alpha-MyHC-reactive T cells in patients with ICI-associated myocarditis and showed that these cells were sufficient to drive myocardial injury in experimental systems [26]. Consistently, cardiac myosin-specific autoimmune T cells promoted myocarditis after checkpoint inhibition in a complementary mouse model [27]. These findings support antigen-directed autoimmunity rather than nonspecific inflammation alone.

PD-1 and CTLA-4 regulate complementary stages of this response. PD-1 limits effector T-cell activation in peripheral tissues, while CTLA-4 constrains T-cell priming and costimulation. A genetic model combining Ctla4 haploinsufficiency with Pdcd1 deletion reproduced the clinical and histological features of ICI myocarditis, including myocardial infiltration by activated T cells and macrophages, and demonstrated a gene-dose interaction between the two checkpoints [28]. Loss of PD-1/PD-L1-mediated restraint may be particularly relevant in injured myocardium, where inducible PD-L1 expression normally provides local cardioprotection. Together, these observations explain why dual PD-1/CTLA-4 blockade produces greater risk than monotherapy and provide a mechanistic rationale for restoring inhibitory costimulatory signaling with agents such as abatacept in severe disease.

### 4.2. Non-T-Cell Immune Mechanisms

Innate immune cells amplify T-cell-driven myocardial damage. Single-cell and spatial analyses of human samples and experimental models have identified expansion of inflammatory CCR2-positive monocyte-derived macrophages within affected myocardium. These cells participate in reciprocal signaling with activated T cells through interferon-gamma-responsive chemokine pathways, including CXCL9/CXCL10-CXCR3, thereby promoting further leukocyte recruitment and tissue injury [29,30]. This macrophage-rich inflammatory circuit may help explain continued myocardial damage even after the initiating T-cell response and represents a potential therapeutic target. Cytokines such as interferon-gamma, tumor necrosis factor, interleukin-1, and downstream chemokines are therefore best viewed as components of an interconnected cellular network rather than isolated mediators.

Humoral mechanisms are less well defined. A pilot autoantibody-profiling study found distinct circulating autoantibody signatures in patients with ICI-associated myocarditis, supporting possible B-cell involvement; however, the small sample size and absence of functional validation preclude conclusions about causality [31]. Likewise, complement can amplify antibody-mediated and innate myocardial inflammation in conventional autoimmune myocarditis, but direct evidence that complement activation is a central driver of ICI-associated myocarditis remains limited. Thus, B cells, autoantibodies, and complement should currently be considered plausible contributors or modifiers rather than established primary mechanisms.

### 4.3. Vascular Toxicity and Accelerated Atherosclerosis

Immune checkpoints also contribute to vascular homeostasis. In atherosclerosis-prone mice, interruption of PD-1 signaling increased lesional T-cell accumulation, macrophage infiltration, inflammatory cytokine expression, and plaque burden, indicating that the PD-1/PD-L1 axis restrains plaque inflammation [32,33]. In clinical cohorts encompassing multiple cancer types, ICI exposure has been associated with an increased rate of atherosclerotic cardiovascular events and accelerated progression of total and noncalcified plaque [34]. Mechanistically, checkpoint blockade may reactivate plaque-resident effector T cells, enhance interferon-gamma-dependent macrophage activation, increase endothelial activation, and weaken anti-inflammatory immune regulation, potentially reducing plaque stability in susceptible patients. These vascular effects are distinct from acute myocarditis and may emerge over a longer time horizon.

The melanoma-specific interpretation requires caution. The shared-clonotype observation was derived directly from patients with melanoma receiving nivolumab plus ipilimumab [25], and melanoma cohorts are highly exposed to dual-checkpoint blockade. By contrast, most alpha-MyHC, macrophage, autoantibody, and vascular data arise from preclinical models or mixed-tumor populations. These pathways are therefore biologically relevant to melanoma care but should not yet be regarded as melanoma-specific mechanisms.

## 5. Clinical Cardiovascular Assessment Before and During ICI Treatment

**Figure 3:**
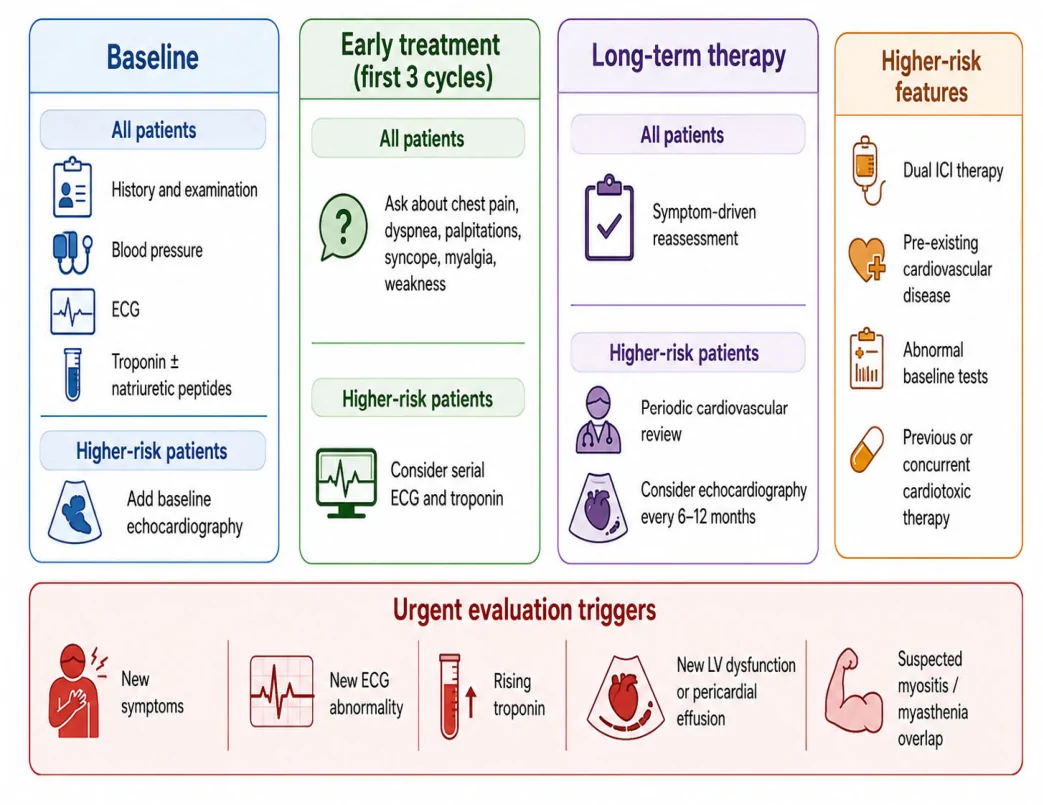
**Proposed cardiovascular surveillance protocol for patients with melanoma receiving immune checkpoint inhibitors.** According to 2022 ESC guidelines, the figure outlines a risk-adapted monitoring strategy for low- and high-risk patients, including cardiovascular assessment, ECG, transthoracic echocardiography, cardiac troponin, and natriuretic peptides at baseline and during follow-up. Figure adapted from ESC 2022 guidelines on cardio-oncology.

Due to the risk of cancer therapy-related cardiovascular toxicity (CTR-CVT), cardiovascular risk assessment before initiating ICI therapy is recommended, although the strength of recommendations varies across guidelines. No prospectively validated model is currently available to predict individual susceptibility to ICI-associated cardiovascular toxicity. A baseline cardiovascular history, physical examination, and optimization of modifiable cardiovascular risk factors should therefore be undertaken in all patients before ICI initiation. The 2022 European Society of Cardiology (ESC) guidelines on cardio-oncology give a class I recommendation for baseline electrocardiogram (ECG), cardiac troponin (cTn), and natriuretic peptides in all patients before starting ICI treatment. The 2025 American College of Cardiology (ACC) concise clinical guidance uses softer language, stating that these tests “can be considered” [35,36]. A proposed risk-adapted cardiovascular surveillance protocol for melanoma patients receiving ICIs is outlined in Figure 3. The following high-risk factors should be assessed: dual ICI therapy, combination with other cardiotoxic antitumor therapies, ICI-related non-cardiovascular events, previous cancer therapy-related cardiac dysfunction (CTR-CD) and pre-existing cardiovascular disease (CVD). High-risk patients should undergo baseline echocardiography, which may also be considered in all patients. Serial ECG and serial cTn assessment should be considered before subsequent ICI doses to detect subclinical cardiovascular toxicity. If long-term ICI treatment (>12 months) is needed, cardiovascular assessment every 6 to 12 months is recommended in high-risk patients and may be considered in all patients. Cardiac evaluation includes physical examination, blood pressure measurement, natriuretic peptides (BNP or NT-proBNP), lipid profile, HbA1c, and ECG. In high-risk patients and in those with elevated baseline cardiac troponin, regular echocardiography may be considered. Of note, in the JAVELIN Renal 101 biomarker substudy (avelumab plus axitinib in renal cell carcinoma), no association was found between elevation in cardiac biomarkers and incidence of MACE (major adverse cardiac events), although assessment was limited by the small number of events. This finding, while derived from a different cancer type and ICI regimen, highlights the ongoing uncertainty regarding the clinical value of routine cardiac biomarker surveillance during ICI treatment [2,35,36,37]. Except where melanoma-specific data are explicitly cited, these surveillance recommendations derive from guidelines and cohorts encompassing multiple tumor types and are extrapolated to patients with melanoma on the basis of treatment regimen and cardiovascular risk.

## 6. Clinical Presentation

ICI-associated myocarditis most frequently develops early after treatment initiation but may occur at any stage of therapy and represents the best-characterized and most severe cardiovascular phenotype. Clinical presentation is heterogeneous and ranges from asymptomatic biomarker elevation to chest pain, fatigue, dyspnea, palpitations, syncope, acute heart failure, high-grade atrioventricular block, ventricular arrhythmias, cardiogenic shock, or cardiac arrest (Table 1).

A preserved left ventricular ejection fraction (LVEF) does not exclude clinically significant myocarditis, and electrocardiographic abnormalities may be subtle or nonspecific. Figure 2 displays the broader spectrum of irCAEs. Among these, myocarditis is the most severe and life-threatening manifestation. Because the presentation may resemble acute coronary syndrome, pulmonary embolism, Takotsubo syndrome, sepsis-related myocardial injury, or noninflammatory heart failure, alternative diagnoses should be actively excluded. Particular attention should be paid to concomitant myositis and myasthenia gravis. ICI-associated myocarditis frequently co-occurs with other neuromuscular immune-related adverse events. Regardless of its exact frequency, this overlap is associated with a more severe clinical course and higher mortality, largely driven by respiratory-muscle involvement [36,38,39]. Patients should therefore be assessed for myalgia, proximal weakness, ptosis, diplopia, dysphagia, and respiratory-muscle involvement. Respiratory failure may result from neuromuscular toxicity rather than cardiac dysfunction alone.

## 7. Diagnostic Approaches

**Figure 4:**
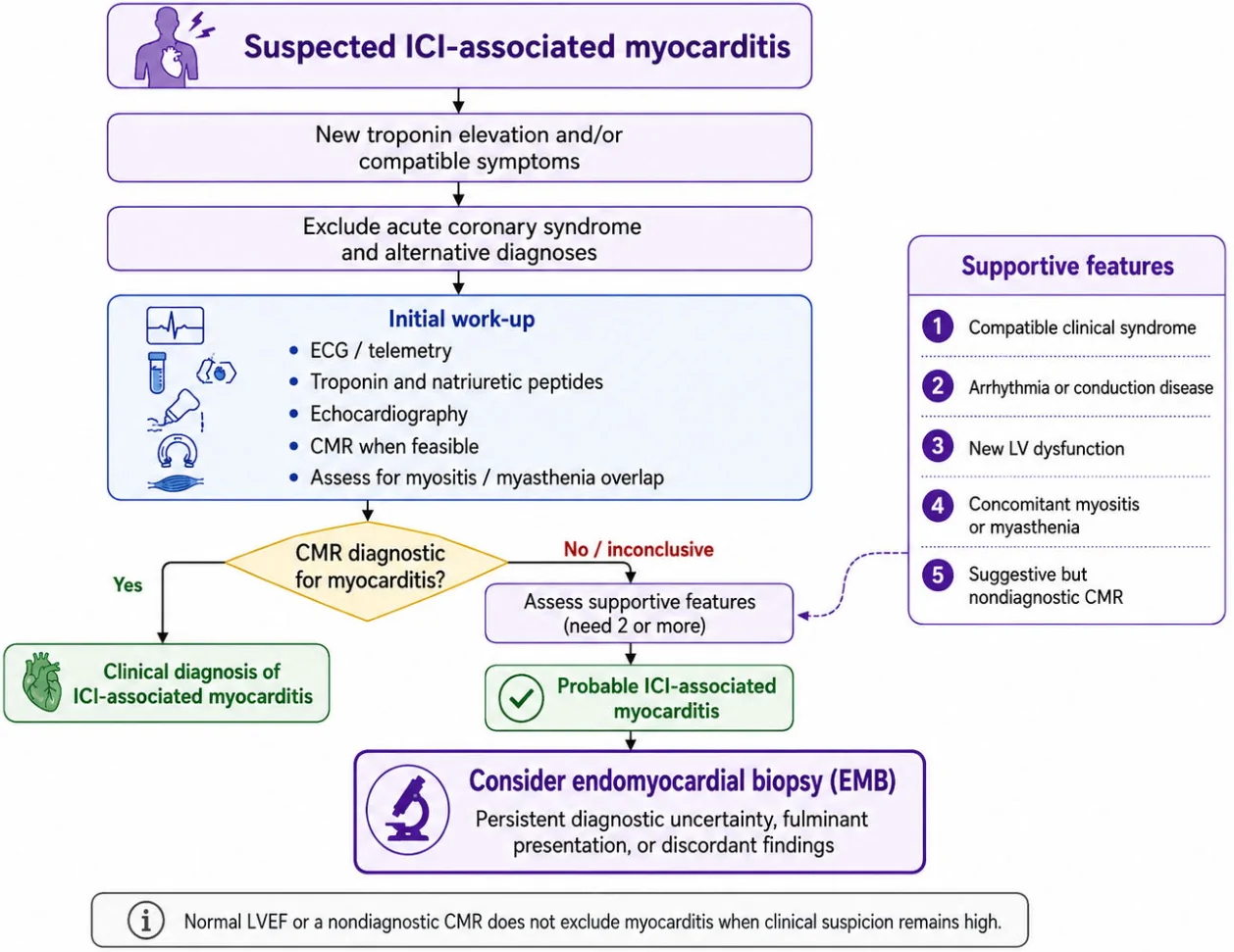
**Practical diagnostic framework for ICI-associated myocarditis.** The figure summarizes a practical diagnostic pathway for suspected ICI-associated myocarditis, integrating cardiac troponin, electrocardiography, CMR, supportive clinical features, and the selective use of endomyocardial biopsy.

Early diagnosis of ICI-induced cardiotoxicity is essential to prevent the progression to life-threatening complications. The 2022 ESC cardio-oncology guidelines recommend baseline ECG and cardiac troponin assessment in all patients receiving ICIs, with baseline transthoracic echocardiography additionally recommended in high-risk patients. Electrocardiography should be performed promptly in any patient with suspected ICI-associated myocarditis. Potential findings include sinus tachycardia, nonspecific ST–T-wave abnormalities, bundle branch block, atrioventricular conduction disease, atrial arrhythmias, ventricular arrhythmias, or diffuse electrical instability. Continuous telemetry is appropriate in hospitalized patients because conduction abnormalities and malignant ventricular arrhythmias may develop during the clinical course. Transthoracic echocardiography provides rapid assessment of left- and right-ventricular function, regional wall-motion abnormalities, global longitudinal strain (GLS), filling pressures, and pericardial effusion. Nevertheless, normal ventricular systolic function does not rule out myocarditis. A reduction in global longitudinal strain may reveal subclinical myocardial dysfunction when the ejection fraction remains preserved. Holter monitoring or implantable loop recorders may be used in patients with suspected arrhythmias, particularly those with recurrent or unexplained syncope. Suspected acute myocarditis is considered in patients with compatible clinical presentation together with ECG findings, elevated cardiac biomarkers, or new ventricular dysfunction. Additional tests may be performed: coronary angiography, autoimmune serological testing, and viral polymerase chain reaction (PCR) when clinically indicated, and positron emission tomography (PET) when CMR is unavailable. CMR is the preferred non-invasive imaging modality for diagnosing myocarditis and should be performed within 2 weeks of symptom onset. According to the updated Lake Louise Criteria, CMR provides strong evidence of acute myocardial inflammation when at least one T2-based criterion of myocardial edema and at least one T1-based criterion of myocardial injury are present. T2-based criteria include a regional or global increase in myocardial T2 relaxation time or increased signal intensity on T2-weighted imaging, whereas T1-based criteria include increased native myocardial T1, increased extracellular volume, or late gadolinium enhancement in a non-ischemic distribution [40]. Importantly, endomyocardial biopsy remains the gold standard for histopathologic confirmation. Diagnostic findings include multifocal inflammatory-cell infiltrates accompanied by overt cardiomyocyte loss. Because biopsy is invasive and carries a small but relevant procedural risk, it is generally reserved for patients with persistent diagnostic uncertainty, a nondiagnostic CMR despite high clinical suspicion, fulminant presentation, unexplained ventricular dysfunction, malignant arrhythmias, advanced conduction disease, or clinical deterioration in which tissue confirmation may alter management. The overall diagnostic approach proposed in this review, integrating the 2022 ESC Cardio-Oncology Guidelines and the 2025 ACC Clinical Guidance, is summarized in Figure 4.

## 8. Surveillance and Management

Cardiovascular surveillance during ICI therapy should be tailored to baseline cardiovascular risk, treatment regimen, and the development of new symptoms or abnormalities. Before ICI initiation, cardiovascular history and examination, ECG, and cardiac troponin assessment are recommended, while natriuretic peptides may also be considered. Baseline echocardiography, preferably including global longitudinal strain, is particularly appropriate in patients with pre-existing cardiovascular disease, abnormal baseline findings, or planned combination ICI therapy [2,35,36]. A practical surveillance framework for patients receiving ICIs is summarized in Table 2.

Baseline biomarker assessment is particularly important because abnormal values may already be present before treatment. In the recent JAVELIN Renal 101 biomarker analysis, cardiac troponin T and NT-proBNP were elevated at baseline in 19.8% and 32.8% of evaluable patients, respectively. Therefore, subsequent biomarker changes should be interpreted in relation to individual baseline values. Although early on-treatment elevations were not significantly associated with MACE, the analysis was limited by the small number of cardiovascular events, including only a few myocarditis cases. These findings support a baseline-referenced and clinically integrated rather than biomarker-only surveillance strategy [37]. When ICI-associated myocarditis is suspected, the first clinical steps should occur in parallel. New chest pain, dyspnea, palpitations, syncope, unexplained fatigue, or proximal muscle weakness during ICI therapy should prompt same-day assessment. Further ICI administration should be withheld; ECG, serial cardiac troponin, natriuretic peptides, creatine kinase, renal and liver function tests should be obtained; and transthoracic echocardiography and early cardiology/cardio-oncology consultation should be arranged. Acute coronary syndrome, pulmonary embolism, infection, and other causes of myocardial injury should be considered concurrently, while patients should be screened for associated myositis and myasthenia-like features. Patients with significant symptoms, dynamic biomarker elevation, ECG abnormalities, ventricular dysfunction, arrhythmias, or conduction disturbances should undergo hospital admission with continuous monitoring. Cardiac magnetic resonance should be performed when clinically feasible, and endomyocardial biopsy may be considered in severe presentations or persistent diagnostic uncertainty. In hemodynamically unstable, fulminant, or otherwise high-risk presentations, high-dose intravenous corticosteroids should be started promptly without waiting for cardiac magnetic resonance or biopsy confirmation [2,35,36]. These recommendations are based predominantly on pan-cancer evidence and expert guidance; their application to melanoma is particularly relevant because dual-checkpoint blockade is used frequently in this disease.

**Figure 5:**
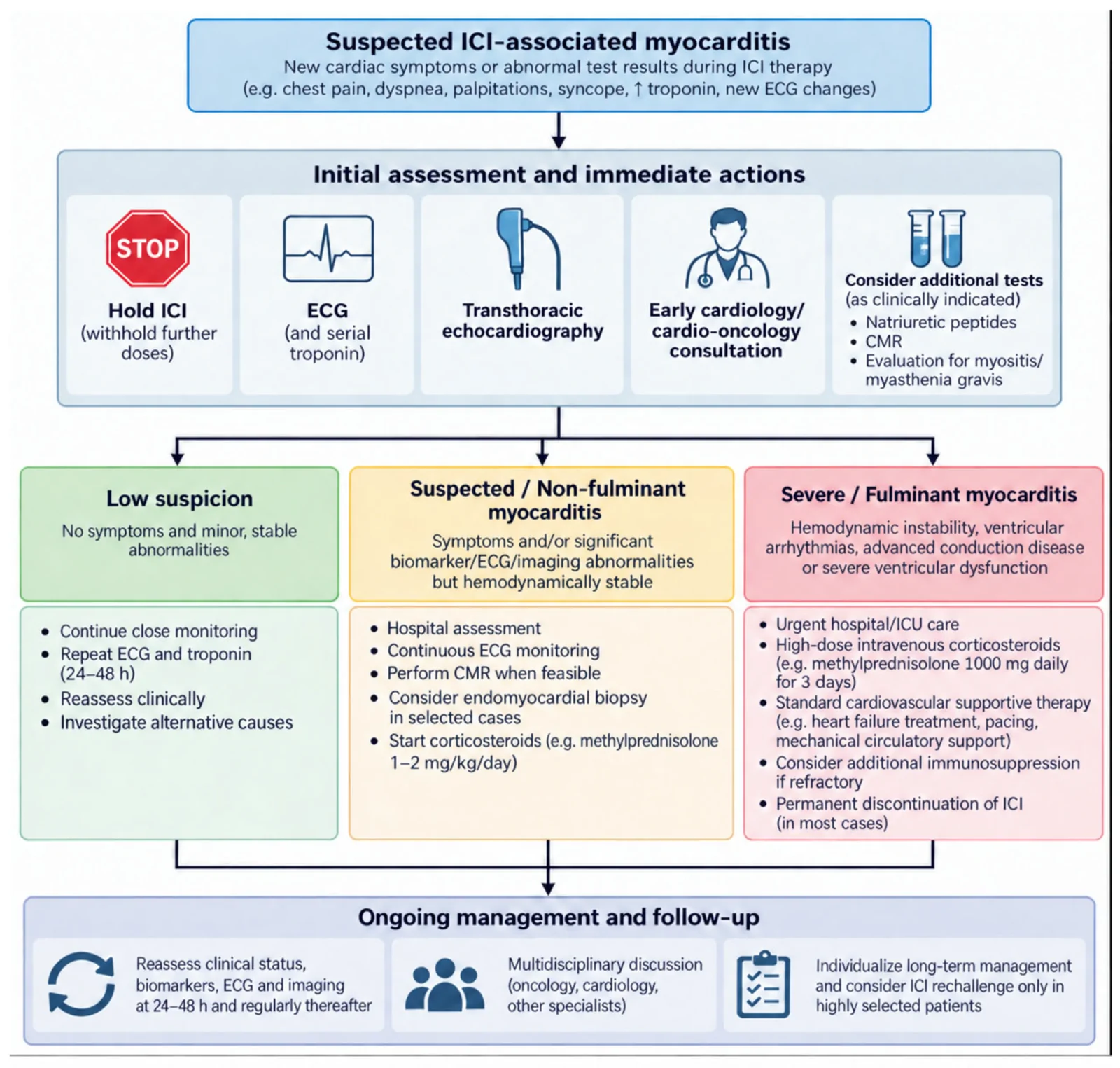
**Proposed algorithm for cardiovascular management of ICI-associated myocarditis in patients with melanoma.** When clinically significant ICI-associated myocarditis is suspected, ICI therapy should be withheld, and the patient should undergo urgent cardio-oncology evaluation, hospital admission, serial cardiac troponin measurements, electrocardiographic monitoring, echocardiography, and assessment for neuromuscular overlap. In severe or fulminant presentations, ICI discontinuation and early administration of high-dose intravenous corticosteroids, preferably within 24 h of symptom onset, remain the principal initial interventions. A commonly used regimen is intravenous methylprednisolone 1000 mg daily for 3 days, followed by a gradual taper over approximately 4 to 6 weeks according to clinical response and biomarker trends.

In severe or fulminant myocarditis, high-dose intravenous corticosteroids should be initiated promptly, commonly with methylprednisolone 1000 mg daily for 3 days, followed by a gradual taper according to clinical response and biomarker trends. Patients with persistent cardiac troponin elevation, hemodynamic deterioration, refractory arrhythmias, or worsening ventricular function should be considered for additional immunosuppression, including abatacept, mycophenolate, tacrolimus, ruxolitinib, intravenous immunoglobulin, or plasma exchange according to clinical severity and institutional expertise. ICI rechallenge should generally be avoided after severe or fulminant myocarditis and considered only in highly selected patients after multidisciplinary risk–benefit assessment [2,35,36]. A practical algorithm for the cardiovascular management of ICI-associated myocarditis, from initial suspicion to escalation and follow-up, is summarized in Figure 5.

Patients with isolated asymptomatic cardiac troponin elevation and no additional evidence of myocarditis may not require immediate high-dose intravenous corticosteroids, but they should be monitored closely with repeat biomarkers, electrocardiography, and clinical reassessment. In contrast, persistent cardiac troponin elevation, hemodynamic deterioration, refractory arrhythmias, worsening ventricular function, or recurrence during steroid taper should raise concern for steroid-refractory myocarditis. Additional immunosuppressive strategies may include abatacept, mycophenolate, tacrolimus, ruxolitinib, intravenous immunoglobulin, or plasma exchange. Selection should be individualized according to disease severity, associated immune-related adverse events, and institutional expertise. Infliximab should be avoided in ICI-associated myocarditis, as it has been associated with higher rates of cardiovascular death in this population and carries a known risk of exacerbating heart failure. Arrhythmias, heart failure, shock, and pericardial complications should be treated according to standard cardiovascular practice, including temporary pacing or mechanical circulatory support when indicated [35,36,41]. For noninflammatory cardiovascular events, continuation or interruption of ICI therapy should be individualized according to event severity, oncologic benefit, and the availability of alternative treatment. For patients who develop arrhythmias, standard antiarrhythmic therapies should be initiated, and consideration should be given to the placement of a pacemaker or implantable cardioverter-defibrillator (ICD) in cases of persistent atrio-ventricular block or life-threatening arrhythmias. Arrhythmias such as atrial fibrillation may occur not only due to intrinsic cardiac involvement but also due to extrinsic causes including thyroiditis, pericardial disease and systemic inflammatory syndromes. ICI treatment may be continued after individualized multidisciplinary assessment when myocarditis has been excluded. [2,35]. Finally, ICI rechallenge remains controversial. Permanent discontinuation is generally recommended after myocarditis requiring corticosteroid treatment, particularly following severe or fulminant presentations. Rechallenge may be considered only in highly selected patients with asymptomatic biomarker elevation or low-severity disease, complete or near-complete recovery, no effective alternative anticancer treatment, and a favorable multidisciplinary risk–benefit assessment. If treatment is resumed, dual-checkpoint blockade should generally be avoided, and close biomarker, electrocardiographic, and imaging surveillance is required.

## 9. Long-Term Outcomes and Prognosis

The prognosis of ICI-associated cardiotoxicity depends primarily on disease severity and the timeliness of diagnosis and treatment. Early studies reported mortality rates of 25% to 50% for ICI-associated myocarditis, whereas more recent large registries have shown lower short-term mortality, likely reflecting earlier recognition of milder cases. Outcomes remain particularly poor in patients with cardiogenic shock, malignant arrhythmias, high-grade conduction disease, depressed LVEF, marked cardiac troponin elevation, or myocarditis–myositis–myasthenia overlap. Long-term follow-up is required to detect persistent ventricular dysfunction or recurrent arrhythmias. Reduced GLS may provide additional prognostic information, even when LVEF is preserved. ICI rechallenge should generally be avoided after severe or fulminant myocarditis and considered only in carefully selected patients after multidisciplinary evaluation.

## 10. Risk Mitigation and Future Directions

Major evidence gaps remain in risk prediction, surveillance, and treatment. Existing data do not establish whether routine biomarker screening improves outcomes, which patients would derive the greatest benefit from surveillance, or how long monitoring should continue. Melanoma-specific risk models are needed to disentangle tumor biology from treatment selection, particularly the frequent use of nivolumab-ipilimumab. The preventive role of conventional cardioprotective therapies, including beta-blockers, renin-angiotensin system inhibitors, and statins, has not been established for ICI-specific toxicity.

Current biomarkers are useful for detecting myocardial injury but lack sufficient specificity for early diagnosis or risk stratification. Candidate approaches include immune-cell phenotyping, cytokine signatures, autoantibodies, circulating microRNAs, and tumor-cardiac antigen profiling. Mechanistic studies should also define the contribution of B cells, macrophages, and other immune effectors beyond the established T-cell paradigm. Prospective studies are required to compare immunosuppressive regimens, define treatment-response endpoints, and determine whether targeted strategies such as abatacept-based therapy can control cardiac inflammation without materially reducing antitumor efficacy [42,43,44,45].

## 11. Strengths and Limitations of This Review

Despite growing interest in immune checkpoint inhibitor-associated cardiovascular toxicity, the evidence base remains limited and heterogeneous. Most available data derive from retrospective observational studies, pharmacovigilance databases, registries, and case series, while prospective studies specifically addressing cardiovascular toxicity in patients with melanoma remain scarce. This heterogeneity affects estimates of incidence and risk, as well as the evidence supporting surveillance and therapeutic strategies.

A major strength of this review is its melanoma-focused and clinically oriented approach. It integrates contemporary epidemiological and mechanistic evidence with current cardio-oncology guidance and provides a practical framework for cardiovascular assessment, surveillance, diagnosis, and multidisciplinary management. Particular attention is given to features that are especially relevant to melanoma, including the frequent use of dual immune checkpoint blockade and emerging epidemiological evidence suggesting an increased susceptibility to ICI-associated cardiovascular and muscular toxicity.

Several limitations should, however, be considered. First, as a narrative review, this work does not follow the formal methodology of a systematic review or meta-analysis and is therefore inherently susceptible to selection bias. Second, although the review is specifically focused on melanoma, much of the available mechanistic evidence and many recommendations regarding cardiovascular surveillance, diagnosis, and management derive from preclinical studies, mixed-tumor cohorts, or pan-cancer guidelines rather than melanoma-specific prospective studies.

Accordingly, some of the mechanisms and clinical recommendations discussed herein necessarily represent extrapolations to the melanoma population and should not be considered specifically validated in this setting.

Third, therapeutic recommendations—including corticosteroid treatment, escalation of immunosuppression, and decisions regarding ICI rechallenge—remain largely supported by observational evidence and expert consensus rather than randomized clinical trials.

Finally, the rapidly evolving nature of cardio-oncology and immunotherapy means that ongoing prospective studies and future guideline updates may substantially refine current approaches to risk stratification, surveillance, and management.

Overall, this review provides a clinically oriented synthesis of the best available evidence while explicitly distinguishing melanoma-specific findings from evidence extrapolated from broader oncological populations and highlighting areas in which melanoma-specific prospective data are still needed.

## 12. Conclusions and Future Directions

ICIs have transformed the management of advanced melanoma, but cardiovascular immune-related adverse events remain an important source of potentially preventable morbidity and mortality. Myocarditis is rare, often occurs early, and may progress rapidly despite preserved left ventricular ejection fraction. The frequent use of dual-checkpoint blockade and emerging epidemiologic evidence of melanoma-associated risk justify heightened clinical vigilance in this population. Prompt recognition, immediate interruption of ICI therapy when myocarditis is suspected, early corticosteroid treatment, and coordinated cardio-oncology care are central to improving outcomes. Future prospective studies are needed to develop validated risk prediction models, optimize surveillance strategies, and evaluate targeted immunomodulatory approaches capable of preserving antitumor efficacy while minimizing cardiovascular toxicity.

## Figures and Tables

**Table 1:** Major cardiovascular toxicities associated with immune checkpoint inhibitors in melanoma.

Management Principles	Risk Features	Diagnostic Tools	Timing/Presentation	Phenotype
Hold ICI; hospitalize and monitor; early high-dose corticosteroids; escalation if refractory	Dual ICI therapy; melanoma; pre-existing CVD; overlapping myositis	ECG, cardiac troponin, natriuretic peptides, echocardiography, cardiac magnetic resonance (CMR); endomyocardial biopsy (EMB) in selected cases	Often early; fatigue, chest pain, dyspnea, palpitations; may be fulminant	Myocarditis
Treat rhythm/conduction disorder; exclude myocarditis; multidisciplinary ICI decision	Myocarditis, systemic inflammation, thyroid dysfunction	ECG, telemetry/Holter, biomarkers and imaging to exclude myocarditis	Palpitations, syncope, AF, ventricular arrhythmias, AV block	Arrhythmias/conduction disease
Treatment according to severity; drainage for tamponade	Immune activation; concomitant myocardial involvement	ECG, echocardiography, inflammatory markers; CMR when needed	Pleuritic chest pain, dyspnea, effusion; rarely tamponade	Pericardial disease
Guideline-directed cardiac care; individualized ICI decision	Baseline CVD; concurrent cardiotoxic therapy	Echocardiography, biomarkers, coronary assessment, CMR	Heart-failure symptoms or acute stress-cardiomyopathy phenotype	Non-inflammatory LV dysfunction/Takotsubo syndrome

**Table 2:** Practical cardiovascular assessment and surveillance framework for patients receiving ICIs.

Action If Abnormal	Higher-Risk Patients	Suggested Assessment	Clinical Phase
Clarify abnormalities and optimize cardiovascular disease before treatment where feasible	Add baseline echocardiography; assess prior CTR-CVT/CVD and combination therapy	History, examination, blood pressure, ECG, cardiac troponin and natriuretic peptides	Before ICI initiation
Repeat testing; urgent imaging and cardio-oncology evaluation if myocarditis is suspected	Closer surveillance with dual ICI or other high-risk features	Clinical review; consider serial ECG and cardiac troponin during initial cycles	Early treatment
Escalate according to symptoms, biomarker changes or new ventricular dysfunction	Consider echocardiography and 6–12-month reassessment	Periodic reassessment guided by risk and symptoms	Prolonged therapy
Hold ICI pending evaluation and initiate myocarditis pathway promptly	Hospital monitoring if myocarditis is suspected	Immediate ECG, cardiac troponin, natriuretic peptides, echocardiography; CMR when appropriate	Suspected acute toxicity

## Data Availability

No new data were created or analyzed in this study. Data sharing is not applicable to this article.

## Abbreviations

The following abbreviations are used in this manuscript:

ACC	American College of Cardiology
AF	Atrial fibrillation
alpha-MyHC	Alpha-myosin heavy chain
AV	Atrioventricular
BNP	B-type natriuretic peptide
CI	Confidence interval
CMR	Cardiac magnetic resonance
CTLA-4	Cytotoxic T-lymphocyte-associated protein 4
CTR-CD	Cancer therapy-related cardiac dysfunction
CTR-CVT	Cancer therapy-related cardiovascular toxicity
cTn	Cardiac troponin
CVD	Cardiovascular disease
ECG	Electrocardiogram/electrocardiography
EMB	Endomyocardial biopsy
ESC	European Society of Cardiology
GLS	Global longitudinal strain
HbA1c	Glycated hemoglobin
HR	Hazard ratio
ICD	Implantable cardioverter-defibrillator
ICI	Immune checkpoint inhibitor
IQR	Interquartile range
irAE	Immune-related adverse event
irCAE	Immune-related cardiac adverse event
LAG-3	Lymphocyte-activation gene 3
LGE	Late gadolinium enhancement
LV	Left ventricular
LVEF	Left ventricular ejection fraction
MACE	Major adverse cardiac events
MI	Myocardial infarction
NCCN	National Comprehensive Cancer Network
NT-proBNP	N-terminal pro-B-type natriuretic peptide
PCR	Polymerase chain reaction
PD-1	Programmed cell death protein 1
PD-L1	Programmed death-ligand 1
PET	Positron emission tomography
sHR	Sub-distribution hazard ratio
